# miRNAs in Regulation of Tumor Microenvironment, Chemotherapy Resistance, Immunotherapy Modulation and miRNA Therapeutics in Cancer

**DOI:** 10.3390/ijms232213822

**Published:** 2022-11-10

**Authors:** Kousain Kousar, Tahir Ahmad, Maisa S. Abduh, Balquees Kanwal, Syeda Saba Shah, Faiza Naseer, Sadia Anjum

**Affiliations:** 1 Industrial Biotechnology, Atta Ur Rahman School of Applied Biosciences, National University of Sciences and Technology, Islamabad 44000, Pakistan; 2 Department of Medical Laboratory Sciences, Faculty of Applied Medical Sciences, King Abdulaziz University, Jeddah 21589, Saudi Arabia; 3 Healthcare Biotechnology, Atta Ur Rahman School of Applied Biosciences, National University of Sciences and Technology, Islamabad 44000, Pakistan; 4 Shifa College of Pharmaceutical Sciences, Shifa Tameer e Millat University, Islamabad 44000, Pakistan; 5 Department of Biology, University of Hail, Hail 81442, Saudi Arabia

**Keywords:** miRNAs, immune evasion, tumor microenvironment, immunotherapy, adoptive T cell therapy, oncolytic virotherapy

## Abstract

miRNAs are 20–22 long nucleotide non-coding ribonucleic acid molecules critical to the modulation of molecular pathways. Immune evasion and the establishment of a suitable tumor microenvironment are two major contributors that support tumor invasion and metastasis. Tumorigenic miRNAs support these two hallmarks by desensitizing important tumor-sensitive regulatory cells such as dendritic cells, M1 macrophages, and T helper cells towards tumors while supporting infiltration and proliferation of immune cells like Treg cells, tumor-associated M2 macrophages that promote self-tolerance and chronic inflammation. miRNAs have a significant role in enhancing the efficacies of immunotherapy treatments like checkpoint blockade therapy, adoptive T cell therapy, and oncolytic virotherapy in cancer. A clear understanding of the role of miRNA can help scientists to formulate better-targeted treatment modalities. miRNA therapeutics have emerged as diverse class of nucleic acid-based molecules that can suppress oncogenic miRNAs and promote the expression of tumor suppressor miRNAs.

## 1. Introduction

According to genome-wide transcriptome analysis, only 2% of genome is translated into protein, whereas the remaining 98% of the eukaryotic genome is transcribed into non-coding RNAs (ncRNAs). These ncRNAs are functional molecules but are not translated into proteins. They include transfer RNA (tRNA), ribosomal RNA (rRNA), and regulatory RNAs that include microRNAs(miRNAs) and long non-coding RNAs (lncRNA). miRNAs are non-coding, 20–22 nucleotide-long RNAs endogenous to cells. They are major mediators of post-transcriptional gene regulation and have a central role in the onset and propagation of various diseases [1] In cancer, miRNAs are involved in oncogenesis through the transcriptional activation of oncogenes and transcriptional dysregulation of tumor suppressor genes. miRNAs bind to 3′ untranslated regions (UTR) of target RNAs, alter their expression, and regulate different processes like cell proliferation, differentiation, and apoptosis [2]. miRNAs are highly conserved across species and regulate various functions by interacting with different transcription factors, ribonucleoprotein (RNP) complexes, proteins, and epigenetic modifiers [3]. 

This review summarizes the role of miRNA in immune evasion, the establishment of tumor microenvironment (TME), how miRNA induces chemotherapy resistance, and the positive role of miRNA in immunotherapy efficacy in cancer. The role of miRNA therapeutics in cancer and its different classes are also discussed in this review article.

## 2. miRNA Biogenesis and Role in Regulation of Cellular Metabolism

miRNAs are transcribed by RNA polymerase II in the nucleus into a local stem-loop structure called primary miRNAs (pri-miRNA). These primary miRNAs are then processed into pre-miRNA with a hairpin structure by ribonuclease III family enzyme DROSHA, which associates with DNA binding protein DGCR8 to form a microprocessor complex [4]. Pre-miRNA are then transported to the cytoplasm through nucleocytoplasmic transporter, RAN-GTP dependent dsRNA binding protein called Exportin 5. In the cytoplasm, this pre-miRNA is processed into mature miRNA by RNase III enzyme DICER. This mature miRNA is incorporated into a protein complex called RISC (RNA-induced silencing complex) to function as a guide against target mRNAs [5]. The existence of mature miRNA in the nucleus has also been reported; it is mediated by nucleus-cytoplasm transporters such as importin5. Inside the nucleus, they are found to be involved in post-transcriptional gene modification, transcriptional gene activation, and transcriptional gene silencing through the binding of miRNA with different gene promoter regions, enhancer regions, nascent RNA transcripts, and regulation of epigenetic pathways [6]. It has been reported that miRNAs have a significant role in the regulation of cellular metabolism and has implications in the pathogenesis of many diseases. They have major roles in metabolic pathways such as lipid and glucose metabolism, amino acid biogenesis. and energy metabolism [7]. In tumors cells, miRNAs participate in promoting the Warburg effect. The Warburg effect supports the tumor microenvironment in the following ways: (a) manages the increased demand of ATP synthesis and supports its incorporation in biosynthesis pathways to channelize the uncontrolled proliferation of cells; (b) promotes lactic acid metabolism even in the presence of adequate oxygen to create an acidic tumor through the accumulation of lactate; and (c) permits ROS signaling homeostasis in cancer cells. This facilitates the creation and maintenance of a protumor, acidic, tumor microenvironment that favors metastasis, invasion, and angiogenesis in cancer [8].

## 3. Cancer-Derived miRNAs

miRNAs in the body not only have the dual role of being a tumor promoter and tumor suppressor, but they are also capable of regulating tumor immune surveillance and escape. A group of miRNAs is capable of tumor cell protection from the immunological response by lowering the tumor cell immunogenicity and down-regulating the anti-cancerous immune attack amplitude. Concurrently, another miRNA panel heightens the anti-cancer immunological clearance. The miRNAs that have modulatory roles are called immune-modulatory or im-miRNA. The im-miRNAs that are derived from tumor cells are not only able to target themselves, but also help in the regulation of a variety of components of the immune system such as Myeloid-derived suppressor cells (MDSCs), Regulatory T cell (Treg), Dendritic cells (DCs), natural killer cells. and cytotoxic T lymphocytes through intracellular communication (microvesicles and exosomes) [9].

The following points depict the role of miRNAs in achieving evasion from an immune attack.

Several immune regulatory miRNAs have the ability to target major histocompatibility complex (MHC1) or one or more integrant of antigen processing machinery (APM) (e.g., latent membrane protein (LMP 8, 9 and 10)) and transporter associated with antigen processing (TAP1), thereby altering antigen processing and presentation in cancer cells [10].

Through the overexpression of HLA-G, which is an unquestioned immune tolerant entity and was primarily associated with fetal-maternal tolerance, the loss of a few members of the miR-148 family such as miR-152, miR-148a, and miR-148b lead to the up-regulation of HLA-G expression, which is another supporting event for immune evasion [11].

The escape of tumor cells from the immune response is also due to the down-regulation of NKG2D ligands, UL16-binding protein (ULBP2), and MHC-I chain-related molecules A/B (MICA/B) at the level of post-transcription. Increased expression of miRNAs that target NKG2D ligand lowers its expression and saves tumor cells from the immune attack of natural killer cells (NKs) and cytotoxic T lymphocytes (CTLs) [12].

Altered expression patterns of inhibitory miRNAs result in the up-regulation of immune checkpoint inhibitor PD-L1 level in the tumor cell, which leads to immune evasion [13].

**Figure 1:**
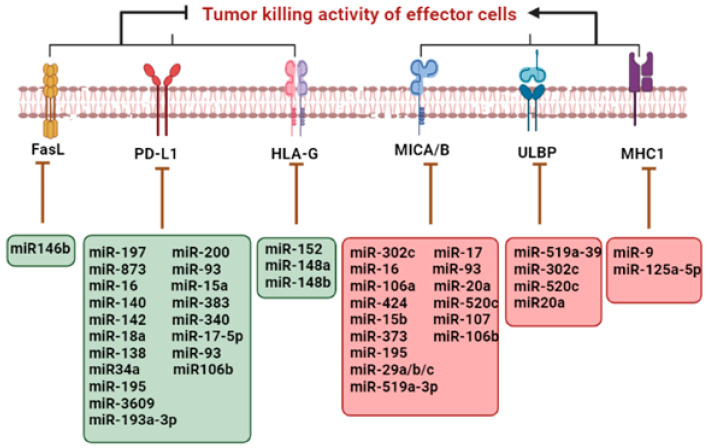
Regulation of immune evasion by cancer-derived miRNAs.

The enhanced expression of death receptor FasL on cancer cells has an inhibitory effect on the immune system as it promotes immune cell death of activated lymphocytes, thus promoting immune evasion. Due to STAT3-induced miR-146b loss, an increased expression of FasL receptor was observed in neutropenia associated with T large granular lymphocyte leukemia [14]. Figure 1 depicts the role of miRNA in immune evasion by the upregulation (green box) and downregulation (red box) of specific receptors.

## 4. Exosome-Derived miRNAs

It has been demonstrated by various studies that immune cells and cancer cells interact with one another through exosomal miRNAs during immune response modulation [15,16]. One akin pathway is an increment in the Tregs population, a CD4+ T cells subset that has a significant self-tolerance maintaining role [17]. It is reported that miR-214 transported to T cells through Lewis lung carcinoma-derived extracellular vesicles cause the down-regulation of PTEN and stimulation of Treg expansion. Thus, tumor cell-derived miRNAs that target immune cells display a significant pathway for tumor immune evasion [18].

An important constituent of tumor-infiltrating immune cells are macrophages that are associated with metastasis. These macrophages are activated by Toll-like receptor (TLR) and interferon-γ ligands for tumor cell elimination. It was reported that miRNAs in the extracellular vesicles (EVs) of the tumor microenvironment are capable of directly activating TLRs. Tumor cells of lung carcinoma release a significant number of extracellular vesicles containing miR-29a and miR-21. These miRNAs are capable of functioning as ligands to TLRs in the nearby cell [19]. The outcomes of these events are the release of TNF-α, IL-6, and other pro-inflammatory cytokines through the NF-kB signaling cascade, which is mediated by the pro-inflammatory response, due to which the tumor microenvironment becomes a pro-metastatic niche. Moreover, tumor-associated macrophages (TAMs) can promote tumor invasion following metastasis. CD4+ T cells released from IL-4 induce alternative activation of TAMs. Higher levels of miR-233 contained by EVs that are released by TAMs can be transported to the tumor cells of the breast, where they stimulate their invasiveness by the regulation of myocyte enhancer factor 2c (Mef2c)-β-catenin signaling cascade. The decrease in Mef2c is linked to β-catenin nuclear accumulation, which stimulates the invasiveness of breast cancer cells. Recent studies have shown that tumor-derived miR-203 can stimulate the monocyte differentiation to M2 macrophages in vivo, which induces metastasis [20].

## 5. miRNA Regulons in Tumor Microenvironment (TME)

miRNAs are very significant in regulating the differentiation and function of immune cells, and their interaction with the microenvironment of tumors. For example, has been reported that miR-27a can alter the presentation of tumor antigens, and a higher level of miR-27a in colorectal cancer tumors decreased the T cell infiltration and was related to poor prognosis and distant metastasis [21]. TME is made up of blood vessels, cellular (stromal and immune cells), and non-cellular (ECM) constituents. The inactivation of T-cells due to higher interactions of PD-L1/PD-1, and CD80/CTLA-4 is caused by the dysregulation of miRNAs. Exosomal miRNAs that are derived from the tumor enhance the macrophages’ polarization from pro-inflammatory (M1) to anti-inflammatory (M2), negatively regulates tumor-associated macrophages (TAMs) phenotype, and cause immune system suppression. The dysregulation of miRNAs also causes the dendritic cells to inhibit the differentiation of T helper cells, which are important for priming antitumor immune response. Some miRNAs are also involved in promoting angiogenesis, inhibiting myeloid-derived suppressor cells, and increasing the activation of cancer-associated fibroblasts, ultimately reprograming the tumor microenvironment [22]. EMT regulatory miRNAs include miR-205, miR-200, miR-9, Let-7, and miR103/107. miR-9 has s significant role in the modulation of tumor microenvironment by interacting with (VEGF)-A and promoting angiogenesis in TME. As reported, miR-9 in breast cancer, induced by MYC and MYCN, increased invasiveness and motility by targeting E-cadherin. This downregulation of E-cadherin by miR-9 promotes angiogenesis by activation of β-catenin pathways, which upregulates VEGF-A expression [23]. On the other hand, it was found that miR-205 stimulates apoptosis by repression of FGF2 and VEGF-A, leading to impairment of PI3K/AKT signaling cascade, which promotes apoptosis. It was found that triple-negative breast cancer cells MDA-MB-231, when transfected with miR-205, exhibited low potential for in vivo metastasis [24].

Lysophosphatidic acid (LPA) presence in the ECM (extracellular matrix) stimulates breast cancer metastasis both in vivo and in vitro. LPA causes the activation of G-protein coupled receptors (LPAR1) which further promotes the miR-21 upregulation by PI3K/ZEB1-dependent pathway in breast cancer cells. Anti-metastatic genes (e.g. PTEN, SPRY2, and PDCD4) were inhibited by miR-21, inducing tumor migration. Varying concentrations of several cytokines in the tumor microenvironment also alter the miRNA expression and activation of several signaling pathways to escape immune surveillance and induce proliferation, metastasis, and tumor invasion [25].

## 6. miRNA as Regulators of Anti-Tumor Response in Cancer

miRNAs play a pivotal role in the positive regulation of anti-tumor immune responses too. These therapeutic miRNAs have been proven to bring positive outcomes by targeting specific molecules, leading to the enhancement of the anti-tumor response in TME, as shown in Table 1. Hepatocellular carcinoma is considered the second deadliest cancer worldly. miR-1294 was downregulated in HCC cells and tissues, whereas Circ_0000854 and IRGQ (immunity-related GTPase Q) were highly expressed. Suppressing circ_0000854 causes regression in HCC cell malignant properties such as cell cycle progression, migration, proliferation, and invasion. miR-1294 suppression relapses the function of circ_0000854 knockdown, whereas Circ_0000854 exerts a sponge effect on miR-1294. IRGQ overexpression revived the miR-1294-induced anti-tumor activity in HCC cells. Animal studies established that by mediating miR-1294 and IRGQ levels in vivo and silencing circ_0000854, metastasis and tumor growth of HCC can be inhibited and can be a source of novel regulatory mechanism for HCC pathogenesis [26].

γδ T cells are a conserved population of lymphocytes. γδ T cells are responsible for antitumor responses through their type 1 inflammatory and cytotoxic effects. The reason is that human γδ T cells upon stimulation with IL-2 or IL-15, which is dependent upon MAPK/ERK signaling, acquire specific active confirmation. A chief modulator of human γδ T cell differentiation is microRNA-181a, which is highly expressed in prostate cancer cells. The high expression of miR-181a is associated with the down-regulation of NKG2D, which is an imperative mediator of cancer regulation. Intriguingly, overexpression of miR-181a hampers the level of NKG2D and TNF-α, and its expression in in-vitro differentiated γδ T cells negatively correlates with an activated type 1 effector profile. In silico analysis identified Map3k2 and Notch2 as miR-181a candidate targets, which was confirmed by luciferase assays coupled with overexpression. These outcomes suggest that miR-181a has a novel role for human γδ T cell differentiation and in next-generation immunotherapies, miR-181a can be a potential manipulator of γδ T cells [27]. 

## 7. Role of miRNA in Establishing Chemotherapy Resistance

One of the prevalent hitches of chemotherapy for cancers such as Gastric Cancer (GS) is multidrug resistance, and, lately, miR-195–5p’s role was studied for amending gastric cancer multidrug resistance (MDR). The outcomes demonstrate that miR-195–5p through Zing finger 139 (ZNF139), generates high sensitivity of oxaliplatin and 5-fluorouracil in multidrug-resistant cells of gastric cancer. In gastric cancer chemoresistance cells, this result proved to be advantageous as it revealed novel channels for better cure responses. Gastric cancer cells’ chemoresistance increases by downregulating the expression of miR-195–5p and overexpression of secretory clusterin (sCLU) [34].

The most major and prevalent form of all renal malignancies is renal cell carcinoma (RCC), which encompasses roughly about 90% of kidney tumors. In renal cell carcinoma patients, miR-195–5p is downregulated and contrarily corresponds with the advanced clinical stage. miR-195–5p directly binds to REGγ, stimulating its role such as cell growth repression, apoptosis induction, and amplifying chemo sensitivity for sorafenib through Wnt/β-catenin pathway [35].

miR-155 and Annexin A2 (ANXA2) are vastly involved in colorectal cancer tissues/cells and analytically have prognostic standards for colorectal patients. miR-155 modified the miR-650/ANXA2 axis and enhanced colorectal cancer advancement and resistance of oxaliplatin through M2 macrophage polarization in colorectal cancer cells [36].

Luteolin, a flavonoid compound, boosts cellular chemosensitivity both in osteosarcomas cells and in xenografts models to cisplatin and doxorubicin. This causes the enhancement of the miR-384 level and downregulation of PTN expression. In addition, PTN expression is directly modulated by miR-384 and could also impede osteosarcoma cells multidrug resistance via PTN/b-catenin/MDR1 signaling axis suppression [37].

HOTAIR, a long non-coding RNA, is involved in the tumorigenesis of cervical cancer and was found to bind with miR-29b. In cervical cancer, a negative correlation existed between miR-29b expression and HOTAIR. In addition, HeLa and Siha cell lines obtained resistance against docetaxel, cisplatin, and paclitaxel facilitated by HOTAIR, whereas in both cervical cancer cell lines, resistance was suppressed by miR-29 against three mentioned chemotherapeutics. Furthermore, epithelial-to-mesenchymal transition (EMT) is enhanced by HOTAIR, whereas an inhibitory effect is exerted by miR-29b. In cervical cancer cells, promoter methylation of PTEN is not affected by miR-29b but by targeting SP1, PTEN expression can be regulated. A major downregulation of PI3K occurs by miR-29b transfection. In cervical cancer, HOTAIR stimulates chemo-resistance by enabling EMT over miR-29b/PTEN/PI3K axis [38].

Acquired chemoresistance of colorectal cancer is a major hurdle in treatment, despite the advancement in proficient chemotherapy. One of the reasons for chemoresistance development is the dysregulation of miRNAs such as miR-199b-3p. Lately, a miR-199b-3p expression was found to be quite altered and upregulated amid cetuximab-resistant and sensitive colorectal cancer cells. Downregulation of miR-199b-3p re-establishes the cetuximab inhibition influence on colorectal cancer cells. In vitro studies suggest that miR-199b-3p silencing could boost anti-tumor properties of cetuximab on cetuximab-resistant colorectal cancer cells and down-regulate CRIM1 through activating Wnt/β-catenin signaling in vivo [39]. Some other miRNAs promoting chemotherapy resistance in different types of cancers are mentioned in Table 2:

### miRNAs as Predictors of Chemotherapy Resistance

The onset of the chemotherapy resistance mechanism emphasized the need to focus on finding indicators that create drug resistance mechanisms. As miRNAs play significant roles in modulating pre and post transcriptional gene modification, they could be the ideal biomarker to analyze and predict chemotherapy resistance mechanisms in patients [50]. Moreover, their stability and ease of detection in biofluids and tissues make them perfect biomarkers to detect primary resistance and acquired resistance against specific targeted therapy in patients [51]. Table 3 shows miRNAs that target anti-VEGF, anti-EGFR, anti-HER2, and immune checkpoint inhibitors in cancer.

## 8. Micro-RNAs in Enhancing the Efficacy of Immunotherapy

### 8.1. Augmentation of Immune Checkpoint Blockade Therapy by miRNA

The most promising therapeutic strategy used against numerous human cancers is the immune checkpoint blockade. miR-140 functions as an integral PD-L1 modulator and is notably decreased in osteosarcoma. Additionally, miR-140 inhibited mTOR signaling and integrated upregulation of miR-140 with mTOR signaling exhibits exceptional synergistic anti-tumor effect [30].

Neuroblastoma (NB) is a complex and fatal neonatological cancer to treat. Neuronal development during embryonic and neonatal stages is integrally interceded by micro RNAs such as miR-15a-5p (miR-15a) and miR-15b-5p (miR-15b). miR-15a/miR-15b act as a tumor suppressor and degrade PD-L1 mRNA by binding to the 3’-untranslated region and RNA-induced silencing complex. In vitro analysis demonstrates miR-15a/miR-15b activated cytotoxicity and NK and CD8^+^T cells activation against neuroblastoma. miR-15a and miR-15b generate an antitumor response by impeding PD-L1 in neuroblastoma [28].

In breast cancer tissues, enhanced miR-21 expression could certainly be interrelated to PD-L1 expression. MiR-21 knock-in mice treated with radiotherapy or anti-PD-L1 antibody resulted in enhanced T and BC cells apoptosis but reduced CD3+CD8+ cells, IFN-γ, serum IL-2, lessened tumor mass and size along with decreased PD-L1 expression. Additionally, in breast cancer cells, PD-L1 expression is enhanced considerably through miR-21 by targeting PDCD4 via PI3K/Akt pathway activation [64]. miR-101 and miR-222 in the tumor microenvironment are capable of regulating cancer-associated fibroblast interactions with the tumor cells. The regulatory role of miRNAs for immune checkpoints is also reported; for instance, the expression of PDL-1 is downregulated in non-small cell lung cancer cell lines because of the direct binding of miR-34 to the 3’untranslated region of PDL-1. This depicts the positive role of miRNAs in predicting the immunotherapy response [65].

In cancer immunotherapy, miR28-influenced T cell exhaustion can provide novel targets for therapeutic markers. miR28 modulates T cell exhaustion by synchronizing immune inhibitory checkpoints like PD1, BTLA, and TIM3, which leads to enhanced expression of TNF-α and cytokine IL-2 [66].

The main hurdle in immune checkpoint inhibitory therapy (ICI) treatment termination is the activation of immune-related adverse events (irAEs). In immune cells, miR-146a (MicroRNA-146a) revealed regulatory roles. IrAE severity can be reduced by therapeutic administration of miR-146a mimic. To prove this finding in humans, 167 patients treated with immune checkpoint inhibitor therapy were selected, and an analysis of the impact of SNP rs2910164 in the *MIR146A* gene on irAE severity was done. SNP rs2910164 resulted in miR-146a expression and progression-free survival reduction, enhanced irAE severity, and amplified neutrophil counts [67].

### 8.2. Role of miRNAs in the Efficacy of Adoptive T Cell Immunotherapy

It is also found in studies that the ectopic expression of miR-200c noticeably increases the CD8+ cytotoxic T lymphocytes (CTLs) antitumor activity during adoptive T cell therapy (ACT) in different mouse models. Cytotoxic T lymphocytes transduced with miR-200c show decreased apoptosis during engrafting and increased persistence in vivo, along with up-regulation of inflammatory cytokine tumor necrosis factor (TNF) and transcriptional regulator T cell factor 1 (TCF1). These changes are elicited by miR-200c by suppression of Zeb1 transcription factor, and, therefore, induce genes that are characteristic of epithelial cells. Overexpression of any one of these genes like *Epcam* is enough to supplement responses of therapeutic T cells against both hematological and solid tumors [68].

miRNAs have the ability to direct T-cell functioning and differentiation. For example, CD4+ T cell (TH1 vs TH2) polarization can be regulated by miR-155. miR-155 knockdown stimulates CD4+ T cells differentiation headed for Th2 phenotype through upregulating the level of its target *c-Maf,* which is a potent IL-4 stimulator transactivator, which eventually results in higher manufacturing of Th2 cytokines IL-4, IL-5, and IL-10. In Th1 cells, the cluster miR-17-92 is overexpressed. The responses of Th1 are controlled by miR-17 and miR-19b through stimulating proliferation, decreasing cell death induced by activation, increasing the production of IFN-γ, and repressing the differentiation of regulatory T cells (Treg). These miRNAs also employ their regulatory role by targeting TGFβRII, CREB1, and PTEN [69]. miR-155 can effectively repress PTPN2 (a tyrosine phosphatase), which downregulates Src Family Tyrosine Kinase (LCK), Src Family Tyrosine Kinase (FYN), FYN Proto-Oncogene, and LCK Proto-Oncogene, leading to the suppression of TCR (T cell receptor) signaling [70]. Another therapeutic microRNA, miR-21 induces T cell activation in a positive feedback loop by down-regulating Sprouty 1 and DUSP10 (serine/threonine phosphatases), both of which inhibit TCR-induced ERK and JNK phosphorylation [71].

### 8.3. miRNA in the Regulation of Oncolytic Virus-Mediated Immunotherapy

miRNA downregulation in cancer cells has been employed as a strategy to provide selectivity to oncolytic virus-mediated immunotherapy in cancer. miRNA response elements specific to miRNAs that are downregulated in tumor tissues have been inserted in 3′UTR of viral genes, making these viruses specific to replicate in tumor cells only and not in healthy cells [72].

The oncolytic measles virus was created by harboring different miRNA target sites in 3′ UTR of H,F,L or N genes of the measles virus. This equipped the virus with targeting sites for miRNAs that are usually suppressed in tumor cells. Depending upon the miRNA, it was found that viral mRNA containing miRNA target sites were degraded or translationally repressed by cognate miRNAs expressed in normal cells but not in tumor cells, demonstrating a mi-RNA-mediated replication control of oncolytic virus both in vitro and in vivo [73].

A miR-21 sensitive oncolytic Herpes virus was created, and its efficacy was established in vitro in cancer cells. An expression construct was created for the dominant-negative (dn) form of HSV replication factor U_L_9, and tandem copies of the miR-21 recognition sequence (T21) were inserted in the 3’ untranslated region. Bacterial Artificial Chromosome (BAC) was used as a vector to introduce the dnU_L_9 construct in the HSV genome with or without the T21 sequence. The virus production and replication capacity of the resulting mutant virus was assessed in both cancer and normal cells. The production of the virus in cancer and normal cells was found to be conditional to the presence of theT21 sequence. This dnU_L_9-T21 virus did not replicate in the absence of miR-21, replicated less efficiently in cells that contained reduced miR-21 activity, and very efficiently in tumor cells [74].

**Figure 2:**
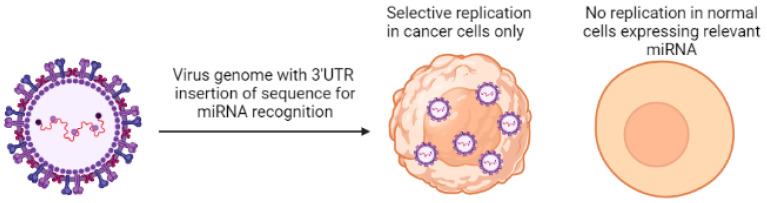
Mechanism of mi-RNA-mediated targeting of oncolytic virus in cancer cells.

Multiple miRNA targeting sites (miRTS) specific against different miRNAs were inserted in a single vector for precise and accurate post-entry targeting of organs during systemic administration of oncolytic virotherapy. miRTS for miR-7, miR-122, and miR-148a, which are abundant in the tissues of major organs like the brain, liver, and gastrointestinal tract, were inserted in measles virus in order to make (MV-EGFP^mtd^) for the purpose of detargeting these tissues during virotherapy. It was found that the oncolytic potential of MV-EGFP^mtd^ was retained in the pancreatic cancer models in vitro and in vivo. This proves that multiple miRNAs deregulated in tumor tissues but expressed in normal cells can be exploited for targeted tropism of oncolytic viruses without compromising their oncolytic potential in cancer cells [75]. Figure 2 shows the mechanism of miRNA-regulated specific targeting of cancer cells by oncolytic virus.

## 9. miRNA Therapeutics in Cancer

miRNA therapeutics aim at targeting, modifying or ideally, reversing the expression of pathological miRNAs. It involves reprogramming or reconstituting intracellular miRNAs in order to enhance the expression of miRNAs that suppress tumor development and functional blocking, the expressional reduction of miRNAs involved in cancer progression [76]. miRNA therapeutics have gained attention because they possess smart pharmacokinetic (PC) properties and improved efficacy or safety as compared to current cancer treatment regimen. These miRNA therapeutics include nucleic acid molecules such as oligonucleotide-based miRNA inhibitors (anti-miRs), miRNA mimics, and recombinant expression vectors carrying miRNA encoding sequences in order to modify the expression of targeted miRNAs. Another currently pursued approach is the use of small molecules that are designed using bioinformatic tools and are identified through experimental screening of pharmacologically active moieties. These are cell permeable and perform their activity by binding with specific miRNA secondary structures or proteins involved in miRNA biogenesis [77]. This strategy was used to identify an inhibitor of miR-21 by screening different low molecular weight chemical compounds. Such inhibitory molecules can also be derived from natural sources; for example, Curcumin targets multiple microRNAs, leading to the inhibition of breast cancer [78,79]. Another class of miRNA therapeutic molecules includes miRNA sponges, which are RNA constructs that harbor multiple sites for RNA binding. They perform their role by sequestering specific cellular miRNAs that are required to be suppressed [80]. Recently, miRNA sponges such as circular RNA are being exploited for cancer treatment. The Circular RNA hsa_circ_0120472, which consists of two RNA binding sites (predicted), has been reported to suppress the development of breast cancer by inhibiting the expression of miR-550a [81]. A latest strategy includes combining miRNAs with conventional cancer drugs. miRNAs together with an anticancer drug can have augmented effect on targeting specific cellular pathways crucial to the inhibition of cancer [82]. As reported, the exosome-mediated delivery of Docetaxel and Cho-miR159 exhibited therapeutic effects in triple negative breast cancer (TNBC) in vitro. On the other hand, in vivo delivery of A15-Exo co-loaded with Dox and Cho-miR159 lead to the silencing of the TCF-7 gene, which has a significant role in antitumor immunity, ultimately leading to the suppression of TNBC tumor without any side effects [83]. 

**Figure 3:**
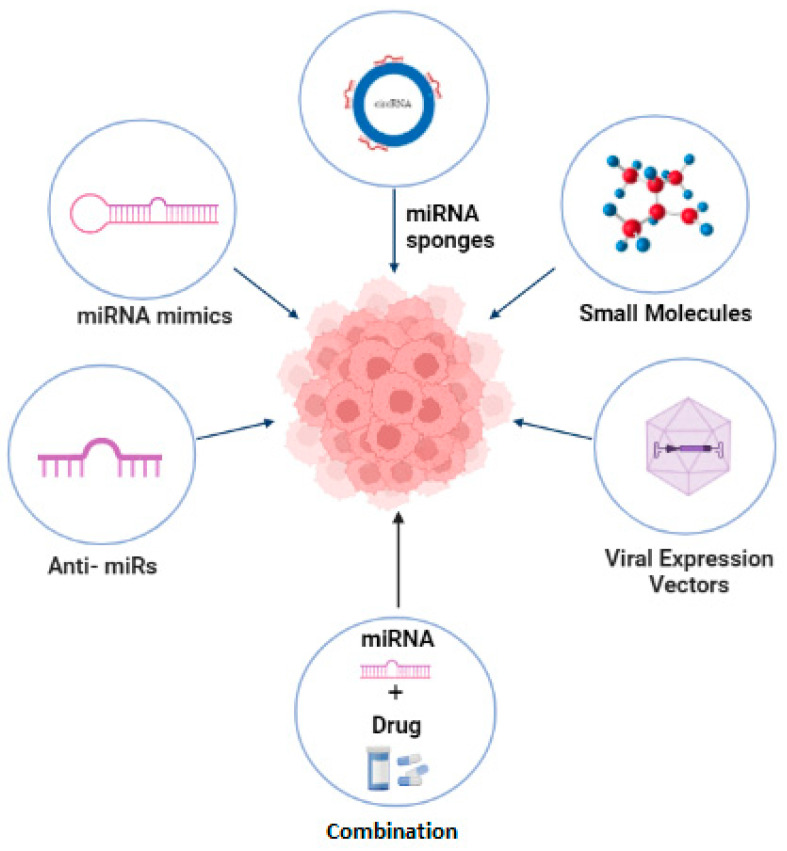
miRNA therapeutics in modulation of tumor associated miRNA expression in cancer.

A stage I clinical trial of MRX34, based on miR-34, reported positive therapeutic outcomes in liver cancer, lymphomas, and melanomas (NCT01829971) [84]. In another phase I clinical trial of Cobomarsen (MRG-106), which is based on miR-155 inhibitor, positive therapeutic outcomes were observed for T cell lymphomas (NCT02580552) [85]. As reported, a phase I clinical trial for MesomiR-1 based on miR-16 depicted better therapeutic outcomes in the treatment of non-small cell lung carcinoma (NSCLC) (NCT02369198) [86]. Figure 3 shows miRNA therapeutic strategies that have a promising role in the modulation of miRNAs in cancer.

## 10. Concluding Remarks

miRNAs are game changers as they have a significant role in the onset, progression, and establishment of TME, immune evasion, and metastasis of cancer. Despite the fact that they are just 20–22 nucleotides long, non-coding ribonucleic acids change the dynamics of gene expression and metabolic pathways through epigenetic modifications and direct silencing of mRNA transcripts. miRNA has a diverse role in diagnosis, prognosis, treatment, and determination of therapeutic efficacy in cancer treatment modalities. Deeper insight and further research into the role of specific miRNAs that support immune evasion, establish chemotherapy resistance, promote TME or even improve antitumor response can pave the way to improved treatment outcomes, early diagnosis, favorable prognosis, disease-free progression, and better modulation of current cancer treatment regimen. Delivery of therapeutic miRNAs to target is a challenge. As a future perspective, therapeutic miRNAs can be delivered to targeted tumors through nanocarriers bearing targeted ligands to augment the therapeutic effect of chemotherapy, radiation therapy, and most importantly immunotherapy, which is currently the most compliant treatment modality for cancer patients. 

## Figures and Tables

**Table 1:** Different tumor suppressor/ oncogenic miRNAs, their associated target molecules, and function with respect to specific solid tumors.

miRNA	Tumor Type Involved	Type of miRNA	Target Molecules of miRNAs Involved in Immune Signaling	Functions Associated	References
miR-1294	Hepatocellular carcinoma	Tumor suppressor	Circ_0000854 and IRGQ	miR-1294 targeted IRGQ and circ_0000854 sponged miR-1294 and IRGQ were targeted to upregulate IRGQ as overexpression of IRGQ restored miR-1294-induced anti-tumor regulation in HCC cells.	[26]
miR-181a	Prostate cancer	Tumor suppressor	NKG2D and TNF-α	MAPK/ERK signaling	[27]
miR-15a and miR-15b	Neuroblastoma	Tumor suppressor	PD-L1	Activation of CD8^+^T and NK cell and cytotoxicity against neuroblastoma in vitro. In vivo studies shows tumor reduction and vasculature.	[28]
miR-301a	Lung adenocarcinoma	Tumor suppressor	Runx3	CD8+ T cell and IFN-γ production enhanced	[29]
miR-140	Osteosarcoma	Tumor suppressor	PDL-1	Increased infiltrates of cytotoxic CD8+ T cells/decreased infiltrates of myeloid-derived suppressive cells and regulatory T cells/mTOR inhibition	[30]
miR-25/93	Breast Cancer	Tumor suppressor	cGAS	NCOA3 causes repression of cGAS during hypoxia	[31]
miR-21	Hepatocellular carcinoma	Oncogenic/Tumor suppressor	PTEN/Akt	CD4+ and CD8+ cell proliferation reduced in oncogenic conditions while in antitumor response CD4+ and CD8+ T cells are activated through PTEN/Akt pathway.	[32]
miR-146a	Hepatocellular carcinoma	Tumor suppressor	STAT3	NK cell dysfunction reverses in vitro while in vivo anti-tumor effect of lymphocytes.	[33]

**Table 2:** Role of different miRNAs in developing chemotherapy resistance in different cancers.

miRNA	Cancer Type	Drug	Target	References
miR-155	Breast cancer	Doxorubicin, Paclitaxel, VP16	FOXO3A	[40]
miR-200c		Trastuzumab	ZEB1, ZNF217	[41]
miR-125 a/b	Colon Cancer	Paclitaxel	ALDH1	[42]
miR-200c		5-fluorouracil	ZEB1/2	[43]
miR-451		SN38, Irinotecan	ABCB1, COX2, ATF	[44]
miR-29b	Cholangiocarcinoma	Gemcitabine	MMP-2	[45]
let-7i	Ovarian Cancer	Cisplatin	PGRMC1	[46]
miR-214		Cisplatin	PTEN	[47]
miR-181a/b	Leukemia	Fludarabine	BCL-2, XIAP MCL-1	[48]
miR-181b			HMGB1, MCL-1	
miR-221/222	Lung Cancer	TKIs, TRAIL	APAF1, TIMP3, PTEN	[49]

**Table 3:** miRNAs targeting anti-VEGF, anti-EGFR, anti-HER2, and immune checkpoint inhibitors in cancer.

Therapy Class	miRNA Involved	Gene Mechanism	Action	References
Anti-VEGF	miR-29b-3p, miR-20b-5p, miR-155-5p	Control signaling of HIF-1α, Akt pathways inhibition	High levels are related to better treatment outcomes of chemotherapy and bevacizumab in patients	[52]
	miR-126	Improve VEGF mediated angiogenic effect	High levels predict resistance towards Bevacizumab	[53]
	miR-455-5p, miR-664-3p	Downregulation of VEGF and neuroligin system	Promising predictive biomarkers of efficacy of Bevacizumab	[54]
Anti-EGFR	miR-345	Dysregulation of EGFR pathway	High levels predict resistance towards irinotecan and cetuximab	[55]
	Let-7	Downregulation of KRAS activity	High levels predict better survival probability in patients harboring KRAS mutation.	[56]
	miR-125b/miR-10	Wnt Signaling pathway	High levels predict Cetuximab resistance	[57]
Anti-HER2	miR-16	Downregulation of AKT through FUBP1 action	Overexpression predicts sensitivity to trastuzumab	[58]
	miR-21	Deregulation of PTEN	High levels predict less sensitivity to trastuzumab	[59]
	miR-494	Reduced FGFR2 expression	Restores sensitivity to Lapatinib	[60]
Immune Checkpoint Inhibitors	miR-152, miR-200b, miR-570	PTEN, DNMT1 targeting	Downregulation leads to increased PD-L1 receptor	[61]
	miR-200	Downregulation of NER-ERCC3/4 pathway	Downregulation leads to increased PD-L1 receptor	[62]
	miR-138-5p, miR-148a-3p	Direct binding with 3’UTR	Overexpression leads to decreased PD-L1 expression (11)	[63]

## Data Availability

Data sharing is not applicable as no dataset was generated during the conception or writing of this review article.
